# Soil properties, rhizosphere bacterial community, and plant performance respond differently to fumigation and bioagent treatment in continuous cropping fields

**DOI:** 10.3389/fmicb.2022.923405

**Published:** 2022-07-22

**Authors:** Jing Xiong, Shuguang Peng, Yongjun Liu, Huaqun Yin, Lei Zhou, Zhicheng Zhou, Ge Tan, Yabing Gu, Hetian Zhang, Jingyi Huang, Delong Meng

**Affiliations:** ^1^School of Minerals Processing and Bioengineering, Central South University, Changsha, China; ^2^Tobacco Research Institute of Hunan Province, Changsha, China; ^3^Beijing Research Institute of Chemical Engineering and Metallurgy, Beijing, China

**Keywords:** continuous cropping barriers, rhizosphere bacterial community, fumigation, bioagents treatment, plant performance

## Abstract

Continuous cropping barriers lead to huge agriculture production losses, and fumigation and biological agents are developed to alleviate the barriers. However, there is a lack of literature on the differences between strong chemical fumigant treatment and moderate biological agent treatment. In this study, we investigated those differences and attempted to establish the links between soil properties, rhizosphere microbial community, and plant performance in both fumigation- and bioagent-treated fields. The results showed that the fumigation had a stronger effect on both soil functional microbes, i.e., ammonia oxidizers and soil-borne bacterial pathogens, and therefore, led to a significant change in soil properties, higher fertilizer efficiency, lower disease infections, and improved plant growth, compared with untreated control fields. Biological treatment caused less changes to soil properties, rhizosphere bacterial community, and plant physiology. Correlation and modeling analyses revealed that the bioagent effect was mainly direct, whereas fumigation resulted in indirect effects on alleviating cropping barriers. A possible explanation would be the reconstruction of the soil microbial community by the fumigation process, which would subsequently lead to changes in soil characteristics and plant performance, resulting in the effective alleviation of continuous cropping barriers.

## Introduction

Long-term monoculture of the same cash crop is a common land use pattern in large-scale agricultural and horticultural intensive production (Tilman et al., 2011). The problems caused by the pattern are referred to as continuous cropping barriers (or obstacles) (Li et al., 2019a). The continuous cropping barriers include many aspects, such as land degradation and plant soil-borne disease infection, which results in crop yield reduction (Zeng et al., 2020). Continuous cropping alters soil physiochemical properties and microbial community, causes soil salinization and acidification, accumulates harmful microbes, reduces fertilizer efficiency, and leads to severe soil-borne diseases (Trabelsi et al., 2012), resulting in yield reduction and huge economic losses in agriculture production (Gentry et al., 2013). Soil microbial communities are closely related to plant nutrient uptake, disease infection (Yang et al., 2017), and immune system and hence, play a crucial role in the formation and alleviation of continuous cropping obstacles (Tan et al., 2021). For example, native root-associated bacteria could inhibit the wilt disease during continuous cropping (Santhanam et al., 2015). Plants also recruit microbes to help them with nutrient acquisition, disease resistance, and stress tolerance (Philippot et al., 2013). However, soil microbiome and their ecological functions, such as biogeochemical process, are affected by the long-term continuous cropping. In a monoculture system, the diversity of the functional group, i.e., ammonia, oxidizers decreases with time, while ammonia oxidation genes (*amo*) are enriched, leading to the rapid oxidation of ammonia to nitrate (Sheng et al., 2013), which resulted in the nitrogen loss and low efficiency of nitrogen nutrients. Previous studies have also revealed that continuous cropping decreased soil bacterial diversity and relative abundance of plant-beneficial microorganisms and increased the abundance of pathogenic *Ralstonia*, which lead to bacterial wilt disease outbreak (She et al., 2017; Xiao et al., 2018). Considering the importance of plant and soil microbiomes in regulating plant productivity (Brooker et al., 2015), it is necessary to study the plant-associated microbial community, such as the rhizosphere microbial community, in continuous cropping field. The importance of soil and rhizosphere microbial community in alleviating continuous cropping barriers has been studied by Zhao et al. (2014). The changes in soil microbial community composition over time were related to changes in the soil nutrient availability during anaerobic soil disinfestation (ASD) treatment to alleviate continuous cropping barriers (Guo et al., 2018). Diverse soil microbial community tended to suppress the soil-borne disease (Yang et al., 2017), while plant growth-promoting rhizobacteria (PGPR) enhanced plant immunity to soil-borne pathogens and increased crop yield in continuous cropping land (Lugtenberg and Kamilova, 2009). Although numerous studies have focused on the role of microbial community in mediating the plant growth and plant health in continuous cropping field, the ecological roles of the rhizosphere microbial community during the alleviation of continuous cropping barriers using biological or chemical approaches have been rarely studied.

Many technologies have been developed to improve soil quality and alleviate the barriers caused by continuous cropping. These include physical, chemical, and biological technologies, such as intercropping (Miao et al., 2011), rotation (Luca et al., 2015; Yang et al., 2016), stereoscopic cultivation (Liao et al., 2018), application of plant beneficial microorganisms, e.g., PGPR (Singh et al., 2017; Goswami and Deka, 2020), and fumigation treatment (Buena et al., 2007; Li et al., 2016, 2019a). Climate and geography, as well as the type of cash crop, however, have limited the agronomic practices of intercropping, rotation, and stereoscopic cultivation (Tilman, 2020). Additionally, the economic returns of the associated plant have also limited the use of these strategies. Therefore, novel methods of efficient and high economic returns are necessary. As an alternative, soil fumigation has been globally employed wherever high-value agricultural crops are subjected to long-term continuous cropping cultures. The fumigants including both biological and chemical have been applied to combat a wide range of pests, including nematodes, fungi, and bacteria, in the soil that have damaging effects on plant growth and crop production (Yagi et al., 1993; Wang et al., 2014). However, fumigation may have adverse health effects on people if not properly administered. Although the fumigation treatment has been effective in alleviating continuous crop obstacle, its application has not been widely adopted (Li et al., 2016). Common and reliable fumigation treatments include chloropicrin (FM1) and dazomet (FM2) (Wang et al., 2013). In addition, soil fumigants, such as chloropicrin, have been shown to affect soil microbial community diversity and composition, change soil nutrient transformation, and affect soil fertility and agricultural productivity (Zhang D. et al., 2019). In this study, soil fumigants were used because of their effectiveness in eliminating continuous cropping barriers. Since microbial communities are among the major obstacles to continuous crop production, several efforts are being made through the use of (micro-)biological techniques (e.g., high-throughput sequencing and high-throughput isolation) to develop beneficial microorganisms to solve the problem of continuous cropping obstacles.

The effects of fumigation or biological agent treatment on continuous cropping problems have been extensively studied. It has been demonstrated that fumigation decreased soil microbial diversity and improved plant growth (Zhang et al., 2020). However, the inherent, complex link between different technologies, underground micro ecosystem, and above-ground plants during alleviating of continuous cropping obstacles is still unclear.

In this study, we employed fumigation and biological agent treatments to alleviate continuous cropping barriers. Rhizosphere bacterial community was investigated through high-throughput sequencing and was linked to plant physiology including phytochrome and growth conditions in different treatments. We compared the effects of biological agent treatments and that of the fumigation treatment, to construct a strong link between microbial community and plant performance. The result of this study will offer theoretical support for developing new approaches for solving the continuous cropping problems.

## Materials and methods

### Experimental design and soil property determination

All field experiments were carried out at the Huayuan Agricultural Science Park, located in Xiangxi, China (109°27′5″ E and 28°24’57″ N). Detailed field experimental design is shown in the Supplementary Material. A field suffering from serious continuous cropping barriers was employed to carry out fumigation and biological agent treatment. The field was divided into 36 equal plots (6 treatments × 6 replicates), and 168 seedlings were planted in each plot. Three fumigation treatments, namely, chloropicrin (FM1), dazomet (FM2), and untreated control (CK_FM), and three biological treatments, namely, two biological agents offered by Prof. Jian Ye from the Institute of Microbiology, CAS, and untreated control (CK_AG). Fumigation and biological treatments were performed before transplanting tobacco seedlings in April 2019. Soil-borne disease investigation, rhizosphere soil sampling, plant sampling, and plant physiological investigation were carried out in August 2019. One sample was collected from each plot by combining rhizosphere soils from 5 plants, resulting in a total of 36 samples. Detailed sampling methods and experimental design are shown in the Supplementary Material (“Materials and Methods” section). Soil pH was determined in deionized water extract (soil/water ratio of 5 g/25 ml) using a pH meter (portable ORP meter, BPH-220, Bell, Instrument Equipment Co., Ltd., China) Organic nitrogen was determined by Kjeldahl digestion; organic carbon was determined by potassium dichromate oxidation-ferrous sulfate titration. Ammonia N and nitrate N were extracted using 2 M KCl and determined with the FIAstar 5000 Analyzer. Available P was extracted with 0.5 M NaHCO_3_ and determined with a spectrophotometer. Available K was extracted using 1 M NH_4_OAc and determined with flame spectrophotometry.

### Phytochrome analysis

The plant pigments were determined using the high-performance liquid chromatography (HPLC)-based method (Schwartz and Von Elbe, 1982). Briefly, a volume of 25 ml of 90% acetone solution was used to extract phytochrome from 0.2 g of liquid nitrogen ground plant leaf material. For extracting the phytochrome, leaf material in acetone solution was ultrasonically crushed for 20 min in an ice bath and then filtered using a 0.45 μm filter membrane. The filtrate was used to measure phytochrome using HPLC. The column used for HPLC was Waters Nova-Pak-C18 (3.9 mm × 150 mm, 4 μm). Analysis condition was set as follows: 30°C, 0.5 ml/min, 5 min for equilibrium, acetone as mobile phase A, and 80% acetonitrile water (V/V) solution as mobile phase A.

### DNA extraction, PCR amplification, and sequencing

Soil total DNA was extracted using Soil FastDNA^®^ Spin Kit (MP Biomedicals, Santa Ana, CA, United States) with 0.5 g of liquid nitrogen ground soil sample, following the manufacturer’s instructions. The V3–V4 region of 16S rDNA was amplified using primer pair 341F (5′-CCTACGGGNGGCWGCAG-3′) and 805R (5′-GACTACHVGGGTATCTAATCC-3′) with 12 bp barcode sequences. PCR amplification was performed on Bio-Rad S1000 (Bio-Rad Laboratory, CA, United States). The PCR reaction included 25 μl 2 × Premix Taq (Takara Biotechnology, Dalian Co., Ltd., China), 1 μl each primer (10 μM), and 3 μl DNA (20 ng/μl) template in a volume of 50 μl. The PCR condition was set as follows: 5 min at 94°C for initialization; 30 cycles of 30 s denaturation at 94°C, 30 s annealing at 52°C, and 30 s extension at 72°C; followed by 10 min final elongation at 72°C. After PCR amplification, the PCR products were extracted using 1% agarose gel electrophoresis, and the products were recovered and mixed in equidensity ratios according to the GeneTools Analysis Software (Version 4.03.05.0, SynGene). The mixed PCR product was purified using the E.Z.N.A. Gel Extraction Kit (Omega, United States). Sequencing libraries were generated using the NEBNext^®^ Ultra™ II DNA Library Prep Kit for Illumina^®^ (New England Biolabs, Ipswich, MA, United States) following the manufacturer’s instructions. The library quality was assessed on the Qubit@ 2.0 Fluorometer (Thermo Fisher Scientific, Waltham, MA, United States). Finally, the library was sequenced on an Illumina Nova6000 platform, and 250 bp paired-end reads were generated (Guangdong Magigene Biotechnology Co., Ltd., Guangzhou, China).

### Data analysis and statistical analysis

FASTQ format and library spliced raw data were obtained from the Guangdong Magigene Biotechnology Co., Ltd., for further analysis. Raw data were processed on the Galaxy pipeline developed by the Institute for Environmental Genomics, The University of Oklahoma^1^ to generate OTU table and represent sequences, as described in our previous studies (Li X. et al., 2017; Gu et al., 2019; Meng et al., 2019). Briefly, the forward and reverse reads were assembled with 10–200 bp overlapping using Flash (Version 1.0) (Magoc and Salzberg, 2011). Lower quality sequences (QC score < 20 and length < 250 bp) were trimmed using BTRIM (Version 1.0) (Kong, 2014), and sequences containing “N” were also removed, and only sequences with 400–440 in length were used for further bioinformatics analysis. Finally, chimeras were removed, and sequences with 97% identity were assigned to the same operational taxonomic unit (OTU) using UPARSE (version usearch v7.01001_i86linux64) (Edgar, 2013), and singletons that have no similar sequences were removed. Sequence number for each sample ranged from 33,953 to 45,904; therefore, we rarefied all samples to 33,953 by randomly choosing sequences. All downstream analyses were carried out using the rarefied OTU table. The raw data were submitted to NCBI SRA database, with the BioProject accession number PRJNA687637. OTU taxonomic assignment was performed by blasting representative sequences to 16S rRNA training set 18 with RDP classifier^2^. Bacterial diversity indexes calculation and beta diversity analyses were performed on the R statistic platform (Version 4.0.3) vegan package (Version 2.5–7). Linear discriminant analysis (LDA) effect size (LEfSe) analysis was carried out to determine the significant difference in the taxa between treatments. Analysis of variance (ANOVA) with the Tukey’s test was performed to identify the significant difference among group means, using aov and TukeyHSD functions on the R software. A *p*-value of less than 0.05 was considered significant. Correlation analysis was performed using the “corrplot” R package with “Pearson” method, and partial least squares path modeling (PLSPM) models were constructed with “plspm” R package.

## Results and discussion

### Fumigants and bioagents treatment effect on plant performance and soil properties

**FIGURE 1:**
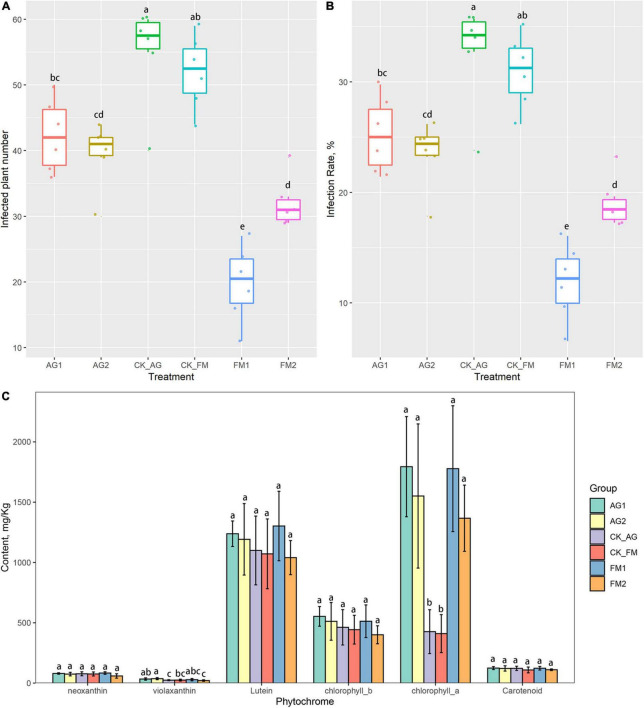
Plant soil-borne disease infection, **(A)** infected plant numbers and **(B)** disease infection rate, and plant phytochrome content in leaves **(C)**. Results are means and SD of six replicated plots; different letters above the columns indicate the differences are significant at *p* < 0.05 level.

Continuous cropping was a leading cause of soil acidification and fertility loss, which resulted in loss in crop yield (Chen et al., 2013), plant physiology, and disease outbreaks (Li S. et al., 2017). Soil properties including pH, AP, AN, NN, and AK were significantly changed by fumigation treatment, particularly, the availability of potassium and phosphorate were significantly increased by both fumigation treatments (Table 1). The increase in plant available nutrients, i.e., potassium and phosphorate, therefore, promoted the growth of plants (Table 2). The plant height, length, surface area of the longest leaf, and number of leaves were significantly increased in fumigation treated fields. The application of bioagents did not cause a significant change in soil properties except for the soil pH, which was increased from 5.90 to 6.16 and 6.29 for AG1 and AG2, respectively. This result is consistent with previous studies, which reported on the increase of available nutrients such as N, P, and K by fumigation (Wang et al., 2014; Li et al., 2019a). Soil nutrient change, particularly changes in available elements (i.e., N, P, and K), indicated that the biogeochemical cycling of NPK was changed by fumigation treatments. For example, the ammonia oxidation tended to be inhibited in fumigation-treated soils because compared with the untreated control soil, there was higher ammonia N content, but lower nitrate N content in fumigation-treated soils. The nitrification ability was demonstrated to be enhanced in long-term continuous cropping and chemical fertilized soils, which is attributed to the enrichment of ammonia oxidizers (Lage et al., 2010; Sheng et al., 2013). Fumigation chemicals have been reported to be effective inhibitors of nitrification (Yan et al., 2017), as many chemicals can act as metal chelators and bind to the active site of the nitrification enzymes such as AMOB subunit (Beeckman et al., 2018). Therefore, one could hypothesize that fumigation may have a strong inhibition effect on soil nitrifiers. Although the biological agent treatment did not cause any change in soil nutrients (N, P, and K contents) or plant performance, it inhibited soil-borne pathogen infection (Figure 1). This may be ascribed to the increased pH as well as the significant change in microbial groups (e.g., beneficial and pathogenic microbial groups) with specific functions in biologically treated soils. However, despite no changes in the microbial community and diversity by the biological agents, the *Aridibacter*, *Arthrobacter, Ralstonia*, and *Ramlibacter* were enriched in AG1-treated field, while *Solitalea* was enriched in AG2-treated field. Additionally, the phytochrome, such as the *chlorophyll a*, was increased by the biological agent treatment (Figure 1). Phytochrome content is essential for crop yield and physiology since the rate of photosynthesis is determined by the phytochrome (Boccalandro et al., 2003). The phytochrome could also regulate the immunity of crops (Moreno and Ballare, 2014). Other than the *chlorophyll a*, the *violaxanthin* was also significantly increased by the biological agent treatment.

**TABLE 1:** Soil properties.

	pH	OM, %	TN, mg/kg	AP, mg/kg	AN, mg/kg	NN, mg/kg	AK, mg/kg
AG1	6.16 ± 0.12ab	4.01 ± 0.79a	1668.4 ± 477.9a	47.78 ± 7.57b	71.95 ± 7.68b	93.74 ± 17.26b	831.7 ± 46.2bc
AG2	6.29 ± 0.13ab	4.53 ± 0.50a	1618.9 ± 270.3a	45.53 ± 13.94b	70.51 ± 10.18b	103.32 ± 10.17ab	823.9 ± 71.2bc
CK_AG	5.90 ± 0.10cd	4.25 ± 0.51a	1252.0 ± 310.7a	35.10 ± 7.51b	71.81 ± 11.06b	95.59 ± 14.84ab	822.2 ± 126.6bc
CK_FM	5.72 ± 0.10d	3.98 ± 0.15a	1530.4 ± 161.4a	46.93 ± 8.15b	71.17 ± 9.37b	126.54 ± 33.37a	673.2 ± 76.1c
FM1	6.05 ± 0.15bc	4.42 ± 0.64a	1493.3 ± 190.4a	64.67 ± 5.40a	124.56 ± 8.86a	54.43 ± 11.40c	1256.4 ± 150.2a
FM2	6.28 ± 0.09a	4.55 ± 0.99a	1471.9 ± 267.8a	66.27 ± 10.03a	115.88 ± 8.67a	50.73 ± 10.83c	966.6 ± 82.1b

OM, organic matter; TN, total nitrogen; AP, available phosphorate; AN, ammonia nitrogen; NN, nitrate nitrogen; AK, available potassium. Results are means and SD of 6 replicates.

Different letters following the results indicate the differences are significant at p < 0.05 level.

**TABLE 2:** Plant physiology properties.

	Height, cm	Stem circumference, cm	Leaf length, cm	Leaf width, cm	Leaf surface area, cm^2^	Leaf number
CK_FM	73.14 ± 2.80b	8.15 ± 0.12a	63.67 ± 3.28c	27.00 ± 0.50a	974.4 ± 117.9c	14.3 ± 0.8b
FM1	97.58 ± 4.65a	7.90 ± 0.34a	75.94 ± 3.69a	28.92 ± 1.51a	1396.1 ± 120.7a	17.4 ± 0.3a
FM2	96.21 ± 6.63a	8.26 ± 0.63a	71.19 ± 2.83b	26.58 ± 1.38a	1204.2 ± 97.9b	17.1 ± 1.3a
CK_AG	68.07 ± 10.27b	7.62 ± 0.90a	60.83 ± 2.03c	27.97 ± 2.06a	1103.6 ± 68.4bc	14.4 ± 1.2b
AG1	72.57 ± 11.07b	8.23 ± 0.64a	62.27 ± 2.90c	27.83 ± 1.14a	1071.5 ± 74.4bc	14.9 ± 1.6b
AG2	69.27 ± 8.94b	7.92 ± 0.43a	61.77 ± 1.79c	27.28 ± 1.40a	1090.5 ± 106.7bc	15.2 ± 1.1b

The leaf length, width, and surface area represent related contents of the longest leaf. Results are means and SD of 6 replicates.

Different letters following the results indicate the differences are significant at p < 0.05 level.

Compared with the biological agent treatment, fumigation treatment had stronger disease inhibition effects. Furthermore, not only did the fumigation treatment increase pH, but it also increased nutrient availability in soil and stimulated crop growth. Previous studies have reported that nutrient availability and plant physiology enhances plant immunity to pathogen infections (Moreno and Ballare, 2014). However, the fumigation only caused a significant increase in chlorophyll a but not in *violaxanthin.* This result indicated that a direct way of alleviating continuous barriers exists through fumigation, i.e., through the direct change in soil properties. However, the use of biological agents, which alter some specific microbial groups and phytochrome, could be an indirect way of alleviating continuous barriers. Similar results were obtained by Singh et al. (2018), who reported an increase in the leaf chlorophyll content in combined fumigation and PGPR-treated fields (Singh et al., 2018). Increase in phytochrome would lead to strong photosynthetic rate and therefore, was responsible for the plant growth promotion (Boccalandro et al., 2003).

### Rhizosphere bacterial community effect on alleviating continuous cropping

**FIGURE 2:**
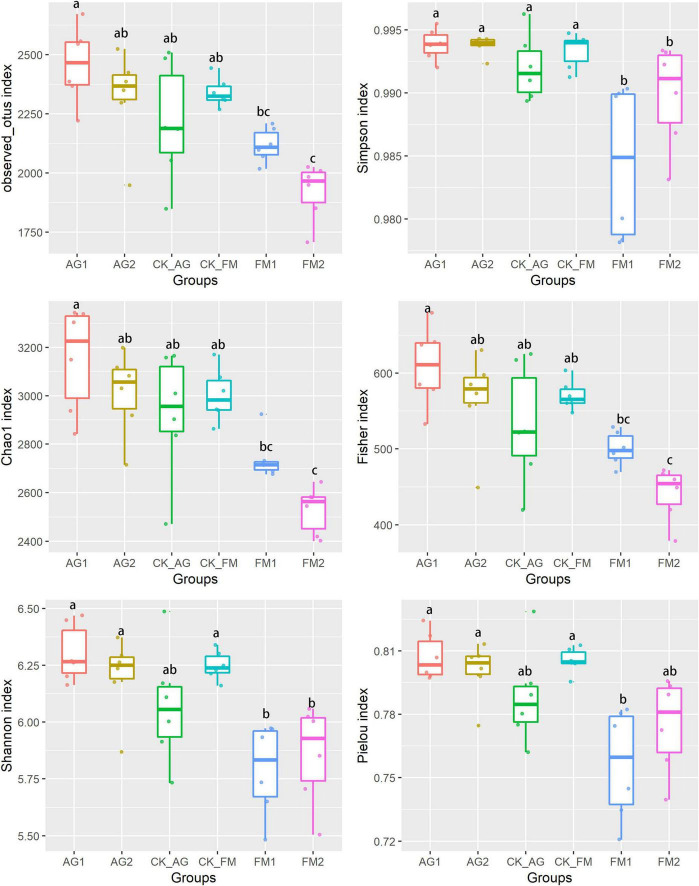
Bacterial diversity index in soil with different treatments. AG1, microbial agent 1 treatment; AG2, microbial agent 2 treatment; CK_AG, untreated control in biological treatment experiment. FM1, CoCl_2_, cobalt dichloride fumigation treatment; FM2, dazomet fumigation treatment; CK_FM, untreated control in fumigation experiment. Different letters above the box indicated the differences between treatments were significant at *p* < 0.05 level.

Considering that the agriculture management patterns (e.g., fertilization and irrigation) were the same for all plots, the changes in soil nutrients, plant performance, and disease infection caused by fumigation treatments could be attributed to the influence of soil microbial community (Li et al., 2019b; Akimbekov et al., 2020). The bacterial community was investigated by high-throughput sequencing of 16S rDNA amplicons. The bacterial diversity was significantly reduced by the application of fumigants (Figure 2), compared with the biological agents, which caused a significant increase in bacterial diversity. This could be explained by the fact that fumigation chemicals are often toxicants to organisms; therefore, microbes that were sensitive to the fumigants were stressed. A significant decrease in bacterial richness (including observed OTU number and Chao1 index) indicated a decline in some bacterial taxa from the soil after fumigation treatment. Ammonia oxidizers might be such a group that was sensitive to fumigation chemicals. This explains the increase in ammonia nitrogen and decrease in nitrate nitrogen following fumigation treatment. Fumigants are well-known poisonous substances used in killing insects, nematodes, and other animals or plants that cause damage to foods, seeds, or human dwelling. Soil fumigants, such as chloropicrin and dazomet, used in this research may kill soil organisms by predation or infection (Yan et al., 2017). It is widely accepted that the diverse soil microbial community would benefit above-ground crops against disease infection and promote plant growth (Brooker et al., 2015; Tao et al., 2017; Yang et al., 2017; Xiao et al., 2018; Mathes et al., 2020). However, this was in contrast to the results obtained in this study for fumigation treatment. This can be ascribed to the fumigation treatment disrupting the entire soil microbial ecosystem (Griffiths et al., 2000), and the crop benefiting from the reassembled rhizosphere microbiome (Nicola et al., 2017; Zhang et al., 2020).

Application of microbial agents in agriculture system has received widespread attention. Previous studies have shown that microbial flora was effective in improving crop yield (Gu et al., 2019; Wang et al., 2020) and also suppressing disease outbreak (Raaijmakers and Mazzola, 2012; Verbruggen et al., 2013; Qin et al., 2019; Wang et al., 2020). Although the biological agent treatments showed some inhibition effects on plant disease, they had less effect on soil bacterial diversity, indicating the disease inhibition effect by biological agents might be a result of other factors such as soil pH, bacterial interactions, and/or plant immunity system.

**FIGURE 3:**
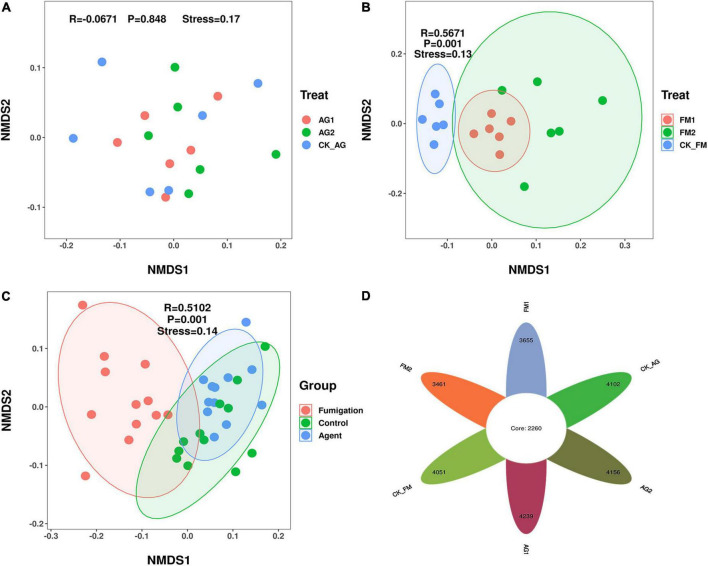
Bacterial community composition in rhizosphere soil after treated with biological agents **(A)** or fumigation chemicals **(B)**. **(C)** An overall look; **(D)** Venn diagram. AG1, microbial agent 1 treatment; AG2, microbial agent 2 treatment; CK_AG, untreated control in biological treatment experiment. FM1, CoCl2, cobalt dichloride fumigation treatment; FM2, dazomet fumigation treatment; CK_FM, untreated control in fumigation experiment.

**FIGURE 4:**
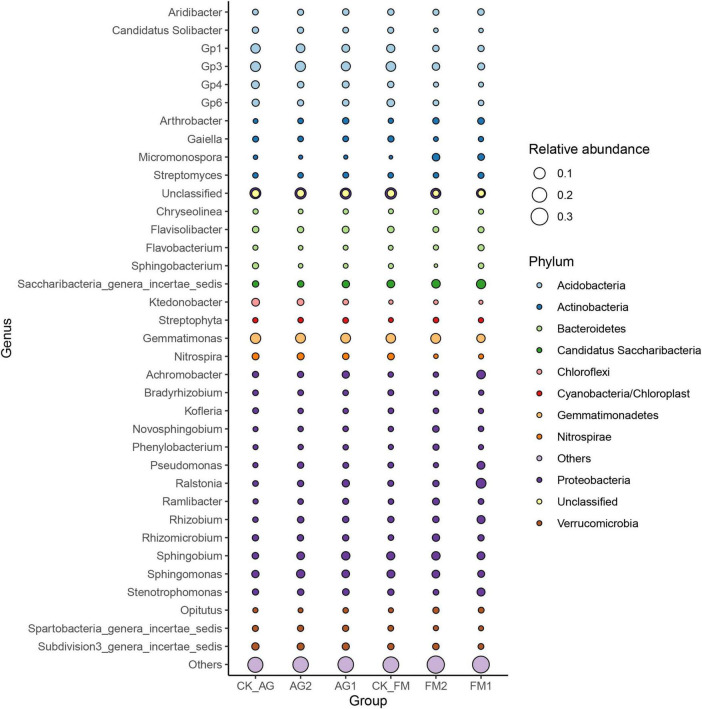
Bacterial community structure at genus and phylum level as indicated by bubble plot. Bubble size represents the abundance (0–1) of each genus, and the color of the burble indicates the phylum. AG1, microbial agent 1 treatment; AG2, microbial agent 2 treatment; CK_AG, untreated control in biological treatment experiment. FM1, CoCl_2_, cobalt dichloride fumigation treatment; FM2, dazomet fumigation treatment; CK_FM, untreated control in fumigation experiment.

**FIGURE 5:**
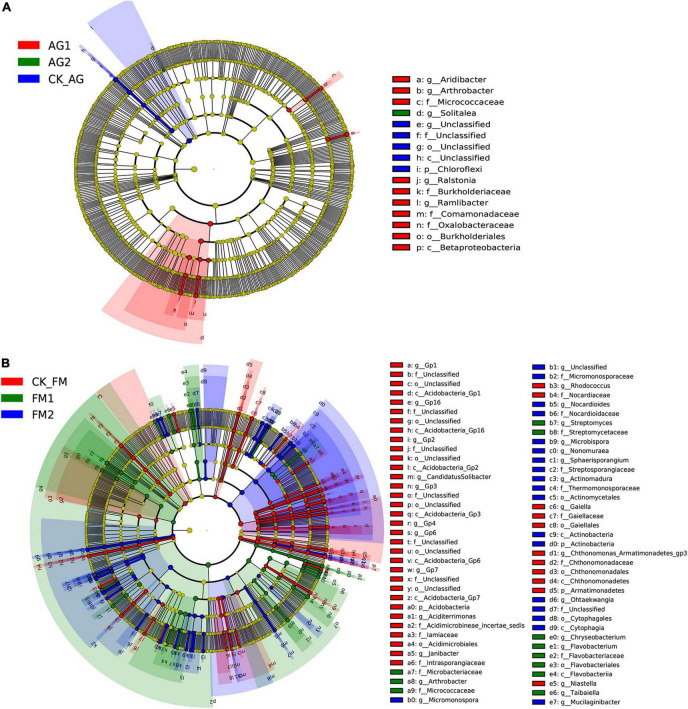
LEfSe analysis showing different taxa among bacterial agent treatments **(A)** or fumigation treatments **(B)**. A taxon with the LDA value of larger than 3.2 was selected as different taxa.

Similar to the changes in bacterial diversity, fumigation had a significant effect on the changes in rhizosphere microbial community composition as compared with the biological agent (Figures 3A–C). Taking together, the Venn diagram showed there were 2,260 core OTUs in all samples (Figure 3D). Compared with the untreated control rhizosphere soil (4,051 for CK_AG and 4,102 for CK_FM), unique OTUs were slightly increased in microbial agent-treated rhizosphere soil (4,239 for AG1 and 4,156 for AG2), whereas the fumigation treatments decreased in unique OTUs (3,655 for FM1 and 3,461 for FM2). The results further confirmed that the fumigation chemicals were toxic to microorganisms. It is worthy to note that, although individual biological agent (Figure 3A) did not cause a significant shift in bacterial community composition, when two agents were combined, they changed the bacterial community composition significantly compared with the untreated control (ADNOIS *F* = 1.83 and *p* = 0.027, based on Bray-Curtis distance). A significant change in microbial community composition and a slight increase in the number of unique OTUs after microbial agent treatment would be responsible for the decrease in soil-borne plant disease infection, as literature reports that the rhizosphere microbial community is closely related to plant health and diverse rhizosphere microbial community would suppress soil-borne diseases (Sanguin et al., 2009; Bakker et al., 2010). Proteobacteria was the most abundant and diverse phylum (averagely 38.63% of the bacterial community) among all 29 phyla in rhizosphere soil, as shown in the bubble plot (Figure 4). *Gemmatimonas* was the most abundant genus, which averagely accounted for 6.35% of the bacterial community, followed by *Gp3* (5.08%), *Gp1* (3.30%), *Sphingobium* (3.22%), *Saccharibacteria_genera_incertae_sedis* (3.03%), and *Sphingomonas* (2.96%). It was also discovered that typical nitrifier, *Nitrospira*, was an abundant genus in rhizosphere soil, and the bubble size indicated the abundance of *Nitrospira* decreased in FM1 and FM2 soil (see also in Supplementary Table 1). We carried out the LEfSe analyses to reveal the specific changes in bacterial taxa after microbial agent and fumigation treatments (Figure 5). Results showed a similar pattern as the change in bacteria diversity and composition. More taxa with significant change (LDA > 3.5) were detected in fumigation treatments than those detected in microbial agent treatments, indicating that fumigation chemicals had stronger effects on soil microbial community than the biological agents. Biological agent AG1 enriched four genera, namely, *Aridibacter, Arthrobacter, Ralstonia*, and *Ramlibacter*. The AG2 only enriched the genus *Solitalea*. Fumigation treatments had an intense effect on rhizosphere bacteria. FM1 treatment enriched *Arthrobacter*, *Streptomyces*, *Chryseobacterium*, *Flavobacterium*, and *Taibaiella*, while FM2 treatment enriched *Micromonospora, Nocardioides, Microbispora, Nonomuraea, Actinomadura, Ohtaekwangia*, and *Mucilaginibacter*. Ordinary rhizosphere microbes such as *Gp1, Gp16, Gp2, Gp3, Gp4*, *Gp6*, and *Gp7* in the Phylum were found to be more abundant in the untreated control. Acidobacteria were more abundant in control soil (Lee et al., 2008). Due to the toxicity of fumigation chemicals to organisms, microbes that lack special strategies to live in extreme conditions die or are inhibited. Previous studies reported that members in the Phylum of *Acidobacteria* are often sensitive to environmental changes, hence, their inhibition by fumigation treatments in this study (Barns et al., 1999). However, it is interesting to note that microbes enriched in fumigation treatment naturally have strong stress-resistant ability, e.g., *Streptomyces* (Thompson et al., 1980), or special structures, such as endospore (e.g., *Microbispora* and *Micromonospora*), that aids them to withstand adverse conditions (Nicholson et al., 2000; Henkin, 2016). Fumigants have been reported to have a strong inhibition effect on soil nitrifiers (Yan et al., 2017); therefore, we selected typical nitrifiers from the OTU table and compared their abundance among fumigation treatments (Supplementary Table 1). The results showed that typical nitrifiers, such as *Nitrospira* and *Nitrospirillum*, decreased significantly in fumigation-treated soil (Supplementary Table 1), and this is consistent with previous reports, which showed the relative abundance of nitrifiers decreased significantly following fumigation treatment (Yan et al., 2017). This could be the reason for the less nitrate nitrogen but more ammonia nitrogen in the fumigation-treated soils.

### The effects of bioagent and fumigation on continuous cropping barriers

**FIGURE 6:**
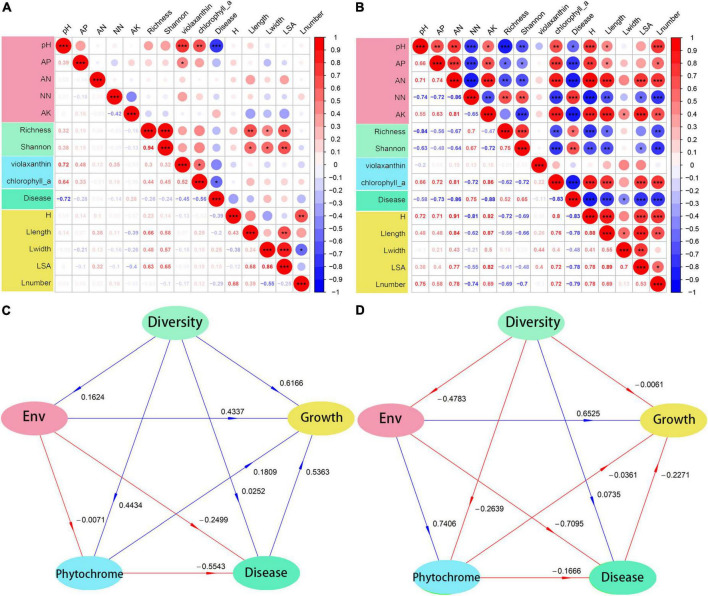
Correlation relationship and PLSPM model of soil physiochemical properties, microbial diversity, phytochrome contents, disease infection and plant growth. **(A,C)** Biological treatment and **(B,D)** fumigation treatment. ENV, soil physiochemical properties. *0.01 ≤ *p* < 0.05; **0.001 ≤ *p* < 0.01; ***p < 0.001.

Fumigation treatment has stronger effect, on soil and plant than biological agents. The correlation analysis (Figures 6A,B) showed a similar trend. Fumigation treatment had a significant effect on either soil properties or rhizosphere microbial community, which led to subsequent changes in other consortia. Therefore, soil properties, microbial community, and plant growth showed significant positive or negative correlations in this study. On the contrary, the consortia in biological treatments showed no correlations.

Soil acidification has been identified as one of the main factors causing soil-borne disease outbreaks; therefore, it was not surprising that the soil pH showed a negative correlation with the plant soil-borne disease infection in both fumigation and bioagent-treated fields. Leaf *chlorophyll a* was negatively correlated with plant disease, and this could be attributed to either disease infection inhibiting the synthesis of phytochrome (Berger et al., 2007) or the synthesis of phytochrome enhancing plant immunity to pathogen infections (Kariola et al., 2005). However, the correlation between bacterial diversity (i.e., Richness and Shannon diversity) and plant growth differed between bioagent-treated fields and fumigation-treated soils. The negative correlation between bacterial diversity and plant growth is in sharp contrast with other studies, which found that plants benefited from the diverse rhizosphere microbial community (Trivedi et al., 2020). The discrepancy between this study’s findings and those of other studies might be explained by the reconstruction of the bacterial community, decreased diversity, change in functional group, and compositional biases following fumigation treatment. This further explains the unusual positive correlation between microbial diversity and disease infection in fumigation treatment. Consequently, to explore the effect of the most important factor on continuous cropping barriers, the PLSPM analyses was constructed to evaluate the direct and indirect effects between indicators and latent constructs (Figures 6C,D). Generally, it was found that bioagent-treated fields had more positive effects than fumigation-treated fields. In bioagent-treated fields, the microbial diversity had a strong direct effect on plant growth (0.6166). On the contrary, microbial diversity had weak direct effects on plant growth (−0.0061) in fumigation-treated fields. However, the effect of microbial diversity on soil properties was weak (0.1624) in bioagent-treated fields but strong (−0.4783) in fumigation-treated fields. The results indicated that the PGPR in bioagent may directly affect plant performance and, therefore, promote plant growth, while fumigation may shift the microbial community that promotes nutrient transformation and plant health and regulates plant growth (Zhang L. et al., 2019).

Agriculture development requires green and sustainable technologies. The need to develop eco-friendly and cost-effective technologies is, therefore, a pressing necessity. This study offers theoretical support for chemical and biological approaches to alleviating the continuous barriers. However, further studies are required to establish the link between agricultural management and soil health, as well as the functional profiles of the soil microbial community. Additionally, the succession pattern of soil microbial community and absolute quantification of soil microbial functional groups needs to be elucidated.

## Conclusion

The effects of bioagent and fumigation on continuous cropping barriers differed in the following three aspects. (i) The effects of bioagent on soil and crop were moderate, as compared with fumigation treatment, which had significantly strong effects on both soil and crop. (ii) Fumigation enhanced soil nutrient availability, decreased rhizosphere microbial diversity, balanced microbial community compositional biases, suppressed soil-borne disease, promoted plant growth, and thus, alleviated continuous cropping obstacles. The bioagents had some effects on soil acidification and, therefore, could effectively suppress soil-borne diseases. (iii) By contrast, the bioagent had strong direct effects, whereas fumigation had an indirect effect on alleviating continuous cropping barriers, accounting for the fact that fumigation first reconstructed soil microbial community, followed by a shift in soil properties and plant performance, resulting in an effective alleviation of continuous cropping obstacles.

## Data availability statement

The datasets presented in this study can be found in online repositories. The names of the repository/repositories and accession number(s) can be found in the article/Supplementary Material.

## Author contributions

JX, SP, ZZ, LZ, and HY conceived and designed the research. JX, YL, GT, HZ, DM, and YG conducted the experiments. SP, YL, and ZZ contributed to new reagents or analytical tools. YL, JH, DM, and YG analyzed the data. DM wrote the manuscript. All authors read and approved the manuscript.

## Conflict of interest

The authors declare that the research was conducted in the absence of any commercial or financial relationships that could be construed as a potential conflict of interest.

## Publisher’s note

All claims expressed in this article are solely those of the authors and do not necessarily represent those of their affiliated organizations, or those of the publisher, the editors and the reviewers. Any product that may be evaluated in this article, or claim that may be made by its manufacturer, is not guaranteed or endorsed by the publisher.

## Footnotes

1
http://zhoulab5.rccc.ou.edu:8080/root

2
http://rdp.cme.msu.edu/classifier/classifier.jsp
