# Supplementary material for: Soil properties, rhizosphere bacterial community, and plant performance respond differently to fumigation and bioagent treatment in continuous cropping fields
Source: Front Microbiol. 2022 Jul 22;13:923405. doi: 10.3389/fmicb.2022.923405 (PMC9354655; doi:10.3389/fmicb.2022.923405)
Supplement: Supplementary file 1 [file Data_Sheet_1.docx]

*Appendix*

Soil properties, rhizosphere bacterial community, and plant performance respond differently to fumigation and bioagent treatment in continuous cropping fields

Jing Xiong^1,4#^, Shuguang Peng^2#^, Yongjun Liu^2^, Huaqun Yin^1,4^, Lei Zhou^3^, Zhicheng Zhou^2^, Ge Tan ^1,4^, Yabing Gu^1,4^, Hetian Zhang^1,4^, Jingyi Huang^1,4^, and Delong Meng ^1,4*^

^1^ School of Minerals Processing and Bioengineering, Central South University, Changsha, 410083, China

^2^ Tobacco Research Institute of Hunan Province, Changsha, 410004, China

^3^ Beijing Research Institute of Chemical Engineering and Metallurgy, Beijing, 101148, China

^4^ School of Minerals Processing and Bioengineering, Central South University, Changsha, 410083, China

^#^ These authors contribute equally

* Correspondence: delong.meng@csu.edu.cn

**2 Material and Methods**

*2.1 Experimental Design*

The experiment was carried out in 2019. All field experiments were carried out at Huayuan Agricultural Science Park, located in Xiangxi China (109°27′5″E and 28°24′57″N). We employed 36 equal plots with six treatments (CK_AG, AG1, AG2, CK_FM, FM1, and FM2) multiply six replicates. Ploughing and ridging were carried out before the field was divided into equal plots (Figure S1). All treatments and replicates were randomly arranged in 36 plots. Each plot has eight rows with 21 seedlings on each row, resulting 168 seedlings in each plot. Agricultural management practices and fertilization regimes were similar in all plots. Seedlings were transplanted in April, 2019. The applied fertilizer consisted of 50 kg/ha special basal fertilizer, 20 kg/ha special top dressing, 15 kg/ha bio-organic fertilizer, and 5 kg/ha hole-applied fertilizer. The ratio of N/P/K (N, P_2_O_5_, and K_2_O) applied was 1:1.2:2.43 (see also in our previous publication [1]). The transplanted crop was irrigated with 300 kg/ha water and 5 kg/ha hole-applied fertilizer. No other pest or disease controls, except for fumigants and bioagents, were applied during the experiment.


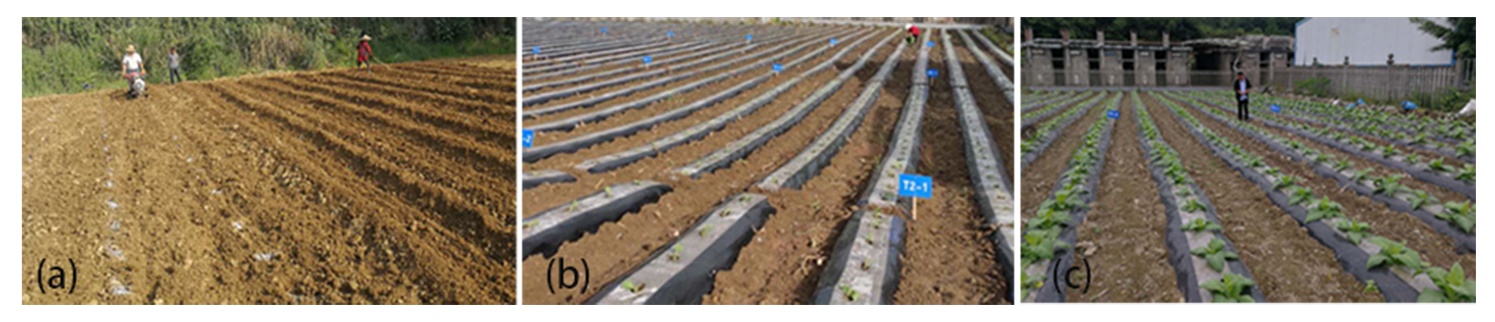


Figure S1 Ploughing and ridging (a), plots (b) and seedling growth (c) in the field.

*2.2 Bioagent and Fumigants*

Before the field experiment, soil sample was firstly collected from the field. The soil sample was brought into laboratory for antagonistic bacteria isolation and pot experiments. Isolated bacteria strains were subjected to plate confrontation assays to test the antagonistic effect against wilt pathogen *Ralstonia solanacearum*. Strains with obvious antagonistic effects were identified by sequencing the 16S rRNA PCR products with 27F and 1492R primer pair. *Chitinophaga* *sp* Ae27 (AG_1) and *Bacillus sp* Gc81 with obvious antagonistic effects against *Ralstonia solanacearum* were selected for the field applications. The microbial strains were isolated and offered by Prof Jian Ye, from Institute of Microbiology, CAS. Prof Jian Ye’s group has done the pots experiment demonstrated that the microbial agents were effective in alleviating the barriers caused by continuous cropping (Data not shown). Therefore, the microbial agents were used in the field experiments.

For biological agent treatment, the microbial agents, together with Chitin as solubilizer was diluted and 300 ml of agent was watered to the root of each seedling immediately after seedlings were transplanted. Fumigation treatments were carried out before filed ploughing. Fumigants (Chloropicrin (FM1) and Dazomet (FM2)) were applied to the field with the amount of 45 g/m^2^, before ploughing. The field was ploughed to mix the soil with fumigants. Fumigation treatments were carried out 30-day before transplanting.

*2.3 Soil sampling and physio chemical property analysis*

Rhizosphere soil sampling, leaf tissue collection, and disease and plant physiology investigation were carried out in August 2019, when the plants were in the maturing stage. In each plot rhizosphere soil from five plants was collected and mixed to obtain an independent soil sample. Rhizosphere soil was collected according to previous publications [2]. Briefly, plants with entire root systems were dig out, bulk soil was shaken off, and rhizosphere soil was carefully collected with the brush. Leaf tissue was sampled by cutting of with scissors. Soil and leaf tissue samples were frozen in liquid nitrogen immediately after collection, and stored in -80 freezer.

Disease infection was investigated by counting infected plants in each plot, and the percentage of infected plants was referred to infection rate (%) [3]. The plants showed at least 5 diseased spots on stem were considered as infected by soil borne pathogens. Plant physiology properties including plant height, length and surface area of the longest leaf, and number of leaves were investigated on 5 plants in each plot, the average value was calculated for each plot.

2.

**3 Results**

Table S1 Relative abundance of nitrifiers in rhizosphere soil after fumigation treatment.

|  | Nitrifiers | *Nitrospira* | *Nitrosospira* | *Nitrobacter* | *Nitrospirillum* | *Nitrolancea* | *Nitrosomonas* |
| --- | --- | --- | --- | --- | --- | --- | --- |
| CK_FM | 2.03±0.68a | 1.77±0.63a | 0.22±0.15a | 0.22±0.15a | 0.0147±0.0081a | 0.0059±0.0037b | 0.0020±0.0036a |
| FM1 | 0.68±0.18b | 0.42±0.18b | 0.17±0.04a | 0.17±0.04a | 0.0054±0.0034b | 0.0231±0.0101a | 0.0201±0.0221a |
| FM2 | 0.56±0.26b | 0.22±0.26b | 0.23±0.09a | 0.23±0.09a | 0.0034±0.0022b | 0.0054±0.0029b | 0.0187±0.0100a |

Reference

1. Xiao, Yunhua, Xueduan Liu, Delong Meng, Jiemeng Tao, Yabing Gu, Huaqun Yin, and Juan Li. "The Role of Soil Bacterial Community During Winter Fallow Period in the Incidence of Tobacco Bacterial Wilt Disease." *Applied Microbiology and Biotechnology* 102, no. 5 (2018): 2399-412.

2. Shi, S., E. E. Nuccio, Z. J. Shi, Z. He, J. Zhou, and M. K. Firestone. "The Interconnected Rhizosphere: High Network Complexity Dominates Rhizosphere Assemblages." *Ecol Lett* 19, no. 8 (2016): 926-36.

3. Yang, Hongwu, Juan Li, Yunhua Xiao, Yabing Gu, Hongwei Liu, Yili Liang, Xueduan Liu, Jin Hu, Delong Meng, and Huaqun Yin. "An Integrated Insight into the Relationship between Soil Microbial Community and Tobacco Bacterial Wilt Disease." *Frontiers in Microbiology* 8 (2017).
